# Blocking autofluorescence in brain tissues affected by ischemic stroke, hemorrhagic stroke, or traumatic brain injury

**DOI:** 10.3389/fimmu.2023.1168292

**Published:** 2023-05-29

**Authors:** Shaoshuai Wang, Xiuhua Ren, Junmin Wang, Qinfeng Peng, Xiaoyu Niu, Chunhua Song, Changsheng Li, Chao Jiang, Weidong Zang, Marietta Zille, Xiaochong Fan, Xuemei Chen, Jian Wang

**Affiliations:** ^1^ Department of Pain Medicine, The First Affiliated Hospital of Zhengzhou University, Zhengzhou, Henan, China; ^2^ Department of Human Anatomy, School of Basic Medical Sciences, Zhengzhou University, Zhengzhou, Henan, China; ^3^ Department of Anesthesiology and Perioperative Medicine, Affiliated Cancer Hospital of Zhengzhou University, Henan, China; ^4^ Department of Epidemiology and Statistics, College of Public Health, Zhengzhou University, Zhengzhou, Henan, China; ^5^ Department of Neurology, The Fifth Affiliated Hospital of Zhengzhou University, Henan, China; ^6^ Department of Pharmaceutical Sciences, Division of Pharmacology and Toxicology, University of Vienna, Vienna, Austria

**Keywords:** autofluorescence, Sudan black B, intracerebral hemorrhage, traumatic brain injury, ICH, cerebral ischemia

## Abstract

Autofluorescence is frequently observed in animal tissues, interfering with an experimental analysis and leading to inaccurate results. Sudan black B (SBB) is a staining dye widely used in histological studies to eliminate autofluorescence. In this study, our objective was to characterize brain tissue autofluorescence present in three models of acute brain injury, including collagenase-induced intracerebral hemorrhage (ICH), traumatic brain injury (TBI), and middle cerebral artery occlusion, and to establish a simple method to block autofluorescence effectively. Using fluorescence microscopy, we examined autofluorescence in brain sections affected by ICH and TBI. In addition, we optimized a protocol to block autofluorescence with SBB pretreatment and evaluated the reduction in fluorescence intensity. Compared to untreated, pretreatment with SBB reduced brain tissue autofluorescence in the ICH model by 73.68% (FITC), 76.05% (Tx Red), and 71.88% (DAPI), respectively. In the TBI model, the ratio of pretreatment to untreated decreased by 56.85% (FITC), 44.28% (Tx Red), and 46.36% (DAPI), respectively. Furthermore, we tested the applicability of the protocol using immunofluorescence staining or Cyanine-5.5 labeling in the three models. SBB treatment is highly effective and can be applied to immunofluorescence and fluorescence label imaging techniques. SBB pretreatment effectively reduced background fluorescence but did not significantly reduce the specific fluorescence signal and greatly improved the signal-to-noise ratio of fluorescence imaging. In conclusion, the optimized SBB pretreatment protocol blocks brain section autofluorescence of the three acute brain injury models.

## Introduction

Fluorescence microscopy has been widely used in animal models and molecular/cellular biology experiments (1). However, significant autofluorescence is frequently observed in tissues, interfering with image analysis and leading to inaccurate results. Many substances cause autofluorescence, such as lipofuscin, porphyrins, collagen, vitamin, and lipoproteins (2). They can interfere with or mask specific fluorescent signals. Without good controls, nonspecific autofluorescence signals can generate a false-positive result and subsequent false conclusion (3) .

Sudan Black B (SBB) is a staining dye widely used in histological studies to eliminate autofluorescence, especially in the rat pancreas (4), mouse liver (5) and kidney (6), and the primate brain (7). However, SBB has not been tested in mouse brain tissue in the context of acute brain injury. Based on our experience, injured brain tissue often exhibits marked autofluorescence, which can obscure specific target signals, leading to false-positive results and incorrect interpretations.

Cyanine-5.5 (Cy5.5) is a standard fluorescent dye widely used to label and track proteins and monitor protein distribution dynamically (8, 9). However, it is unclear whether SBB can mitigate the autofluorescence of injured brain tissues and whether combining the two provides a precise cellular localization and distribution of proteins or Cy5.5-labeled drug-like compounds in the brain.

In this study, we characterized autofluorescence in brain sections of the three models of acute brain disease, including the collagenase-induced intracerebral hemorrhage (ICH) model, the traumatic brain injury model (TBI), and the middle cerebral artery occlusion (MCAO) model for focal cerebral ischemia. Furthermore, we tested the applicability of SBB in a fluorescence labeling protocol and an immunofluorescence staining protocol. Finally, we established a simple and effective protocol to block autofluorescence in the three models tested.

## Materials and methods

### Animals

Ten-week-old C57BL/6 male mice (weight, 24-28g) were obtained from Beijing Vital River Laboratory Animal Technology Co., Ltd [SCXK (Beijing) 2016-0006]. Mice were housed in a pathogen-free environment with 12 h cycles that alternate dark and light, five per cage, a controlled temperature (25°C), and relative humidity (45-55%) with free access to standard food and water. The experimental protocol was approved by the Animal Ethics Committee of Zhengzhou University (ZZUIRB 2022-31) and was performed according to national guidelines. In addition, this study was carried out according to the ARRIVE and RIGOR guidelines for using experimental animals (10, 11). The mice were randomly divided into groups using random numbers generated by a random number website (http://www.randomization.com).

### The ICH model

Our previous publications describe the detailed procedure for modeling ICH by striatal collagenase injection (12, 13). First, mice were anesthetized with isoflurane (4% induction and 2% maintenance). Then, we injected collagenase VII-S (C2399, 0.05U in 0.5 µL sterile saline, Sigma, St. Louis, MO) into the striatum, whose coordinates were 0.8 mm anterior, 2.1 mm lateral of the bregma, and 3.3 mm depth (14–16). After the operation, the mice were replaced in the rest cage until they woke up in a warm environment. At the same time, food was added inside the cage to prevent the mice from losing access to food from the cage rack above due to surgical injury.

### The TBI model

As previously described, we used Feeney's method to establish the mouse weight-drop model of TBI in the right parietal cortex (17, 18). Mice were anesthetized with isoflurane. The scalp was opened 1 mm behind the window of the right parietal bone and 1 mm from the midline, and a 4 mm craniotomy was performed on the skull using a dental drill and trephine. A 20g steel rod was released from a height of 20 cm to induce craniocerebral injury (control depth of 1 mm), and the scalp was sutured.

### The middle cerebral artery occlusion (MCAO) model

We used the MCAO model to induce focal cerebral ischemia (19–21). Briefly, mice were anesthetized with isoflurane. The common carotid artery was then cut and exposed along the midline of the neck. Next, a 6.0 monofilament nylon suture was used to pass through the left external carotid artery, which blocks the left middle cerebral artery, resulting in focal transient cerebral ischemia for 90 minutes.

### Injection of IL-10 conjugated to Cy5.5

Briefly, 25 µg IL-10 (R&D Systems, 217-IL-005) and the fluorescent dye Cy5.5 (Med Chem Express, HY-D0924) were incubated overnight, and the unbound Cy5.5 was removed by centrifugation using a 3 KD ultrafiltration centrifuge. The labeled IL-10 was stored in the dark at -80°C upon use. Then, we injected IL-10 conjugated to Cy5.5 into the bleeding site 24 hours after ICH. The injection coordinate was 0.8 mm anterior, 2.1 mm lateral to the bregma, and 3.3 mm depth.

### Tissue processing

Twenty-four hours after ICH or 72 hours after TBI, the animals were deeply anesthetized with isoflurane and transcardially perfused with preheated 37°C saline until the liver turned white. For the Cy5.5 fluorescent tag experiment, the brains were removed, placed in an embedding box, embedded with OCT, and quickly frozen at -80°C. The brains were then cut into 20 µm coronal sections on a cryostat. The frozen brain sections were fixed in acetone at -4°C for 15 min. The brain sections were dried naturally, then washed with PBS for 10 min. For immunofluorescence experiments, after 37°C saline infusion, paraformaldehyde infusion was used. The brain was taken out and soaked in paraformaldehyde for 12 h and then soaked in a 20% and a 30% sucrose solution, each for 24 h. After the OCT embedding, sections were performed.

For ischemic stroke, we sacrificed the mice 5 days after MCAO. Mice anesthetized with isoflurane were injected with saline and paraformaldehyde successively. Subsequently, the brain tissue was removed and fixed with paraformaldehyde for 24 h. Paraffin sections of brain tissues were obtained using the paraffin embedding method. Paraffin sections were immersed in sodium citrate antigen retrieval solution and heated in a microwave oven at high heat for 3 min and low heat for 8 min. Then, immunofluorescence staining was performed.

### SBB treatment

To prepare the SBB solution, 300 mg of SBB (Sigma-Aldrich, 199664-25G) was left to stir overnight in 100 ml of 70% ethanol in the dark (7). The mixture was filtered using a 0.22 µm filter to remove the undissolved SBB. The solution was stored at 4°C and sealed in an airtight container in the dark until use. The SBB solution was diluted to 0.15% SBB working solution fresh before use. Stock solutions older than 3-4 weeks were discarded to avoid the accumulation of poly azo derivatives that form during prolonged storage (4). At the end of the immunofluorescence or Cy5.5 experiment, SBB treatment was performed. The 0.15% SBB solution was pipetted dropwise onto coronal brain sections to cover the section adequately, and the sections were incubated for 5 min in the dark. The sections were briefly rinsed with 70% ethanol for 30 seconds and then rinsed in PBS for 5 min. The sections were briefly rinsed with 70% ethanol for 30 s and then rinsed in PBS for 5 min. Brain slices were imaged under a fluorescence microscope. The brain slices from the vehicle group were treated with 70% ethanol for 5 min in the dark and then washed with PBS for 5 min.

### Immunostaining

According to our established protocol (22–24), brain slices were blocked in 10% fetal bovine serum at room temperature for 2 hours and then incubated overnight at 4°C with primary antibodies, including rabbit anti-NeuN (neuronal marker; 1:200; Abcam, ab177487) and rabbit anti-Iba-1 (microglial marker; 1:300; Affinity, DF6442). Next, the brain slices were washed three times with PBS, and then the secondary antibody Alexa Fluor 488-conjugated goat anti-rabbit IgG (1:300; Jackson, 156194) was incubated for 2 h at room temperature in the dark. Finally, the brain slices were washed three times with PBS.

### Fluorescence imaging

Brain sections were examined before and after SBB treatment using a fluorescence microscope (Nikon DS-Ri2, Tokyo, Japan) equipped with fluorescein isothiocyanate filters (FITC, 465-495 nm, green), Texas red (Tx Red, 540-580 nm, red) and 4', 6-diamidino-2-phenylindole filters (DAPI, 361-389 nm, blue) filters. Using the NIS-Element BR imaging software, the images were collected and analyzed with a color camera (Nikon, Version 5.01, Tokyo, Japan). The exposure time of each group of images was the same (exposure time for immunofluorescence images: 40x field of vision: 800 ms, 100x field of vision: 600 ms, 200x field of vision: 600 ms; ICH model: FITC and Tx Red exposure time: 2 sec, DAPI exposure time: 100 ms; TBI model: FITC and Tx Red exposure time: 1 sec, DAPI exposure time: 100 ms).

Three or four brain sections per mouse were taken, and four visual fields located near or around the lesion were selected for each brain section. The brain sections were imaged in the untreated condition and again at the same site after SBB treatment. All images were processed with white balance using ImageJ software. The relative intensity of the signal was calculated using ImageJ to determine the average fluorescence intensity in an arbitrary unit compared to the control group.

### Statistical analysis

We performed statistical analysis using GraphPad software (GraphPad Prism 8.4.3; GraphPad Software, Inc., La Jolla, CA). All data were normally distributed as evaluated by Shapiro-Wilk's test. All data are presented as means ± standard deviation (SD). We use the paired t-test to compare the differences between the samples before and after treatment. Furthermore, we used a two-way analysis of variance (ANOVA) test followed by Sidak post-hoc test to compare differences between multiple groups. A value of p<0.05 was considered statistically significant. The results of the normality test are provided in the **Supplementary Materials**.

## Results

### Autofluorescence occurs in brain tissues damaged by ICH

**Figure 1:**
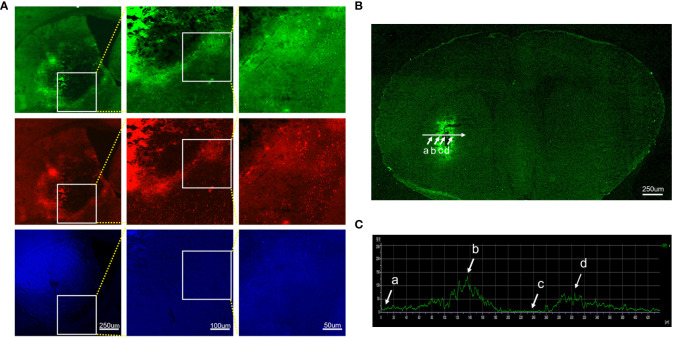
Characteristics and distribution of autofluorescence in brain tissues damaged by intracerebral hemorrhage. Brain sections were observed under different filter systems at high magnifications. The brain tissues around the hematoma showed bright autofluorescence. **(A)** FITC, Tx red, and DAPI filter. **(B)** Autofluorescence distribution of intact brain sections of hemorrhage-affected striatum under a fluorescence microscope, targeting regions of the brain far from the bleeding foci (a), immediately around the bleeding foci (b, d), and within the bleeding foci (c). **(C)** Histogram of the mean fluorescence intensity profile along the selected axis region. The white arrow indicates the corresponding part of the axis in **(B)**.

To investigate whether autofluorescence is present in the ICH-damaged striatum, we examined brain sections under a fluorescence microscope with FITC, Tx Red, and DAPI filters. We observed intense autofluorescence with the above three filters (**Figure 1A**). To characterize the overall distribution of autofluorescence in brain tissues of the ICH-affected striatum, we applied a method in which a horizontal axis (white line) was added to the bleeding site in the image (**Figure 1B**). The change in fluorescence intensity on this axis was quantitatively analyzed (**Figure 1C**). The fluorescence intensity was low in regions of the brain far from the bleeding site (a), increased strongly immediately around the bleeding site (b), was extremely low within the bleeding site (c), and increased immediately around the bleeding site again (d). In general, autofluorescence from ICH-damaged brain tissues is emitted primarily from tissues immediately around hemorrhagic foci. The intensity of autofluorescence is markedly higher than in the rest of the surrounding areas.

### SBB blocks autofluorescence of brain tissues damaged by ICH

**Figure 2:**
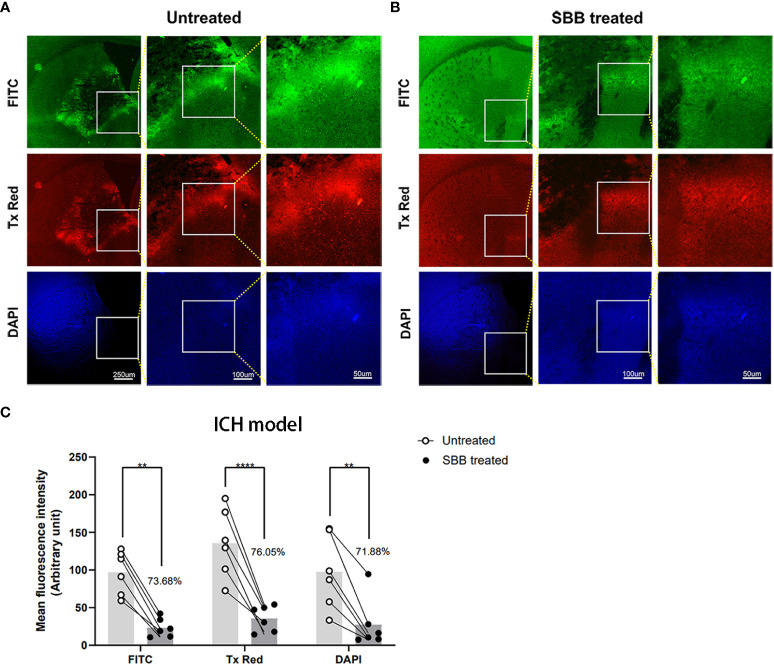
Sudan black B (SBB) blocks autofluorescence of brain tissues damaged by intracerebral hemorrhage. **(A)** Untreated brain sections of the striatum after intracerebral hemorrhage. **(B)** The same brain sections were treated with 0.15% SBB and images were acquired showing the same area around the hematoma **(A)** before and **(B)** after SBB treatment. The images were visualized with three different microscopic filter sets. **(C)** The mean fluorescence intensity under the FITC, Tx Red, or DAPI filter in the same brain sections before and after SBB treatment. The percentage of mean fluorescence of the brain sections relative to the untreated brain sections is quantified in **(C)** Untreated vs. SBB treated: FITC, **p=0.0023; Tx Red, ****p<0.0001; DAPI, **p=0.0037; F_Interaction_=0.6899, F_Time_=2.081, F_Column Factor_=51.23; two-way ANOVA; n=6 mice/group.

To determine whether SBB can block autofluorescence from brain tissues, we compared the intensity of autofluorescence from the same brain region (striatum) in the same brain sections before and after SBB treatment (**Figure 2**). We observed a robust nonspecific fluorescence signal in the untreated group (**Figure 2A**). After SBB treatment, the autofluorescence of damaged brain tissue was significantly removed in all three sets of filters (**Figure 2B**). SBB treatment eliminated tissue autofluorescence considerably by 73.68% (FITC), 76.05% (Tx Red), and 71.88% (DAPI) compared to the untreated level (**Figure 2C**).

### Comparison of IL-10 and NeuN fluorescence signal in brain tissues damaged by ICH before and after SBB treatment

**Figure 3:**
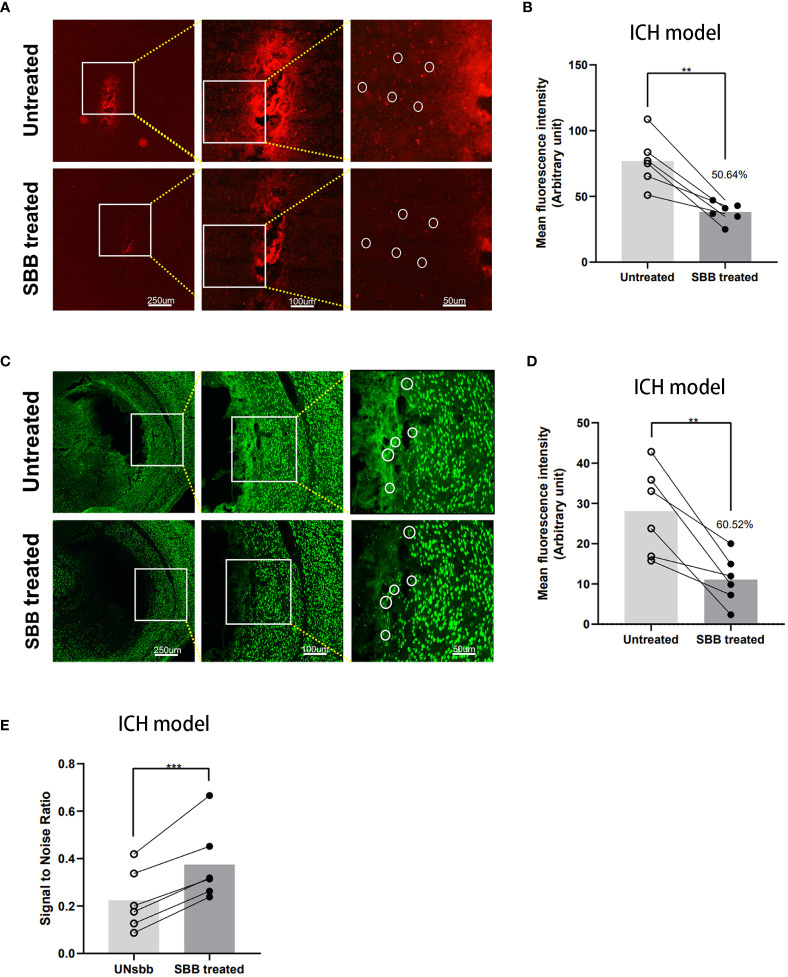
Comparison of the IL-10 and NeuN fluorescence signal of brain tissues damaged by intracerebral hemorrhage before and after Sudan black B (SBB) treatment. **(A)** Striatal injection of Cy5.5-labeled IL-10 was used to evaluate the fluorescence of intracerebral hemorrhage-damaged brain tissues in the same brain sections, before and after SBB treatment, under a fluorescence microscope with the Tx Red filter. The white circles indicate representative regions of the significant difference in fluorescence signal before and after SBB treatment. **(B)** SBB treatment eliminated tissue autofluorescence by 50.64% of the untreated level (**p=0.0029; t=5.416; paired t-test; n=6 mice/group). **(C)** Anti-NeuN antibody labeled with Alexa Fluor 488 was used to assess the intensity of immunofluorescence of brain tissues in brain sections affected by ICH before and after SBB treatment under a fluorescence microscope using the FITC filter system. The white circles indicate representative regions of the significant difference in fluorescence signal before and after SBB treatment. **(D)** SBB treatment eliminated tissue autofluorescence by 60.52% of the untreated level (**p=0.0072; t=4.369; paired t-test; n=6 mice/group). **(E)** Compared to the untreated group, SBB treatment significantly improved the signal-to-noise ratio of fluorescence imaging (***p=0.0007; *t*=7.411; paired t-test; n=6 mice/group).

IL-10 labeled with Cy5.5 was injected into the striatum. Background autofluorescence and specific fluorophore labeling of Cy5.5 were mixed and almost indistinguishable. However, after SBB treatment, the fluorescence signal of Cy5.5 improved markedly (**Figure 3A**), with tissue autofluorescence reduced significantly by 50.64% compared to the untreated level (**Figure 3B**).

NeuN is a specific marker for neurons. To evaluate the effect of SBB on ICH-damaged brain tissues, we used the Alexa Fluor 488 labeled anti-NeuN antibody for immunofluorescence staining with or without SBB treatment. SBB reduced the background signal and highlighted the specific fluorescence signal (**Figure 3C**). The reduction in background autofluorescence was significant (60.52% compared to the control group (**Figure 3D**)). We also analyzed the signal-to-noise ratio before and after SBB treatment, which significantly improved after SBB treatment compared to the control group (**Figure 3E**).

### SBB blocks autofluorescence of brain tissues damaged by TBI

**Figure 4:**
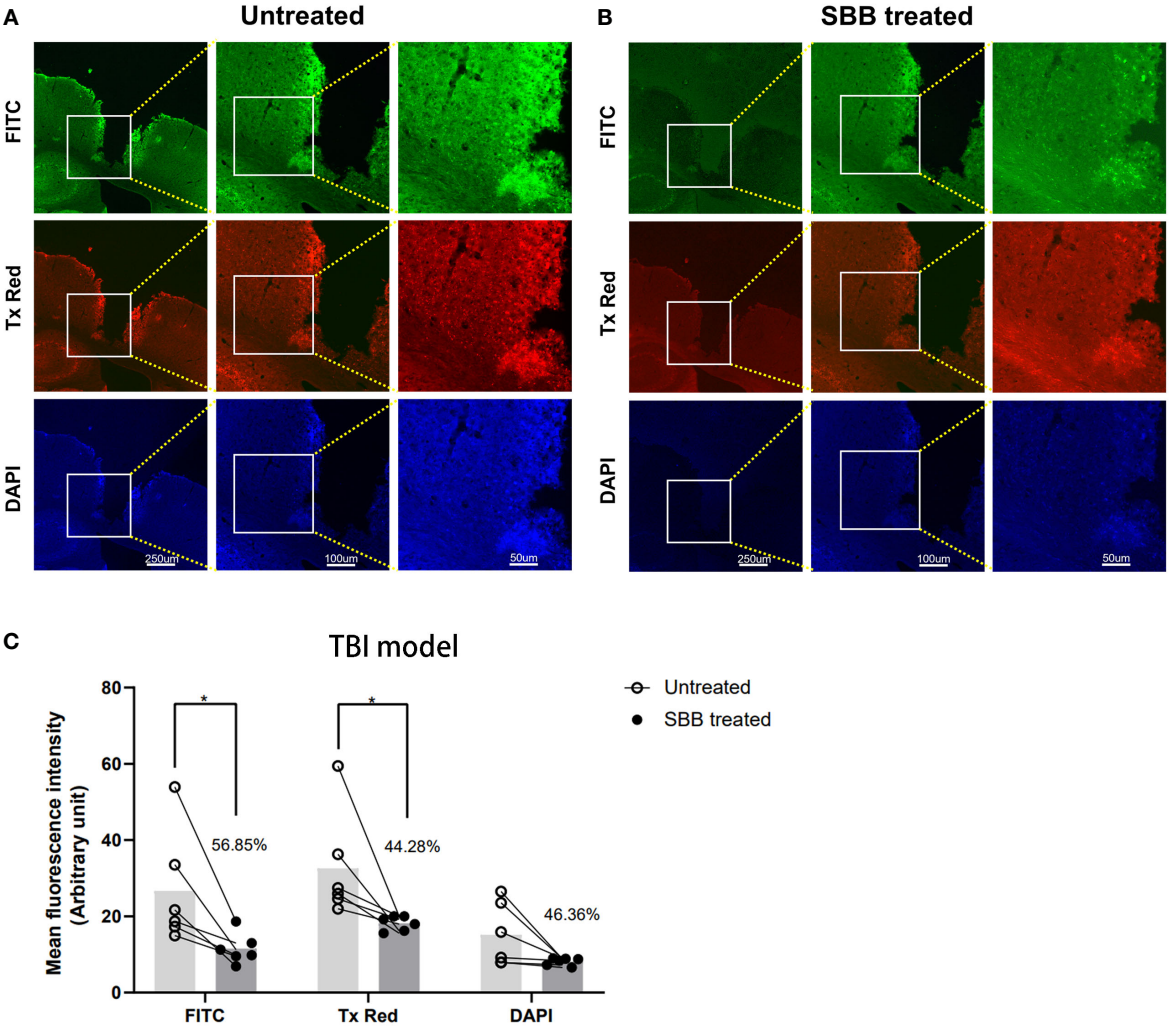
Sudan black B (SBB) blocks the autofluorescence of brain tissues damaged by traumatic brain injury. **(A)** Autofluorescence of brain tissues damaged by traumatic brain injury. **(B)** The same brain section was treated with 0.15% SBB. FITC, Tx Red, and DAPI filters were used to collect images from the same brain area in sections before and after SBB treatment. **(C)** Quantified mean fluorescence intensity of brain sections treated before and after SBB. The percentage represents the decrease in the mean fluorescence intensity after SBB treatment. Untreated vs. SBB-treated: FITC, *p=0.0227; Tx Red: *p=0.0316; DAPI: *p*=0.4786; F_Interaction_=0.7227, F_Row Factor_=6.746, F_Column Factor_=15.92; two-way ANOVA; n=6 mice/group.

We examined brain sections under a fluorescence microscope to determine whether SBB can also block the autofluorescence of brain tissues damaged by TBI. We detected a large amount of autofluorescence in the brain tissues surrounding the lesion in untreated sections (**Figure 4A**). However, after SBB treatment, the autofluorescence of the three channels of the evaluated damaged brain tissues decreased (**Figure 4B**) by 56.85% for FITC, 44.28% for Tx Red, and 46.36% for DAPI, respectively, compared to the untreated group (**Figure 4C**).

### Comparison of Iba-1 immunofluorescence of brain tissues damaged by TBI before and after SBB treatment

**Figure 5:**
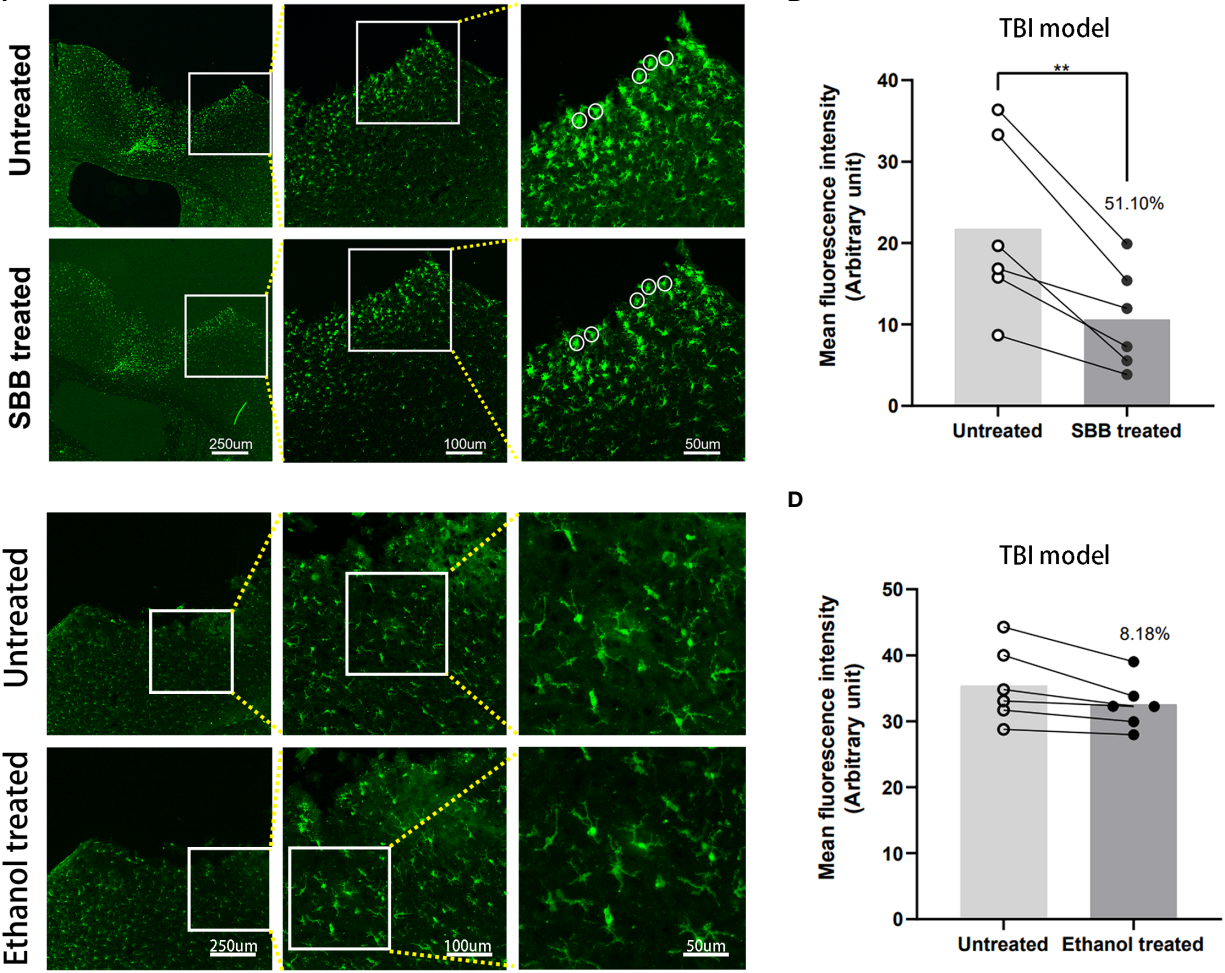
Comparison of Iba-1 immunofluorescence in brain tissues damaged by traumatic brain injury (TBI) before and after Sudan black B (SBB) or ethanol treatment. **(A)** Anti-Iba-1 antibody labeled with Alexa Fluor 488 was used to assess immunofluorescence in brain tissues damaged by TBI in the same brain sections before and after SBB treatment under a fluorescence microscope using the FITC filter. The white circles indicate the representative regions of the significant difference in fluorescence signal before and after SBB treatment. **(B)** Background autofluorescence intensity in SBB-treated brain sections decreased by 51.10% compared to before SBB treatment (**p=0.0054; *t*=4.679; paired t-test; n=6 mice/group). **(C)** Anti-Iba-1 antibody labeled with Alexa Fluor 488 was used to assess the immunofluorescence of brain tissues damaged by TBI in the same brain sections before and after ethanol treatment. **(D)** The background autofluorescence intensity in ethanol-treated brain sections decreased by 8.18% compared to before treatment (*p*=0.1734; *t*=1.587; paired t-test; n=6 mice/group).

Next, we sought to evaluate the efficacy of SBB to enhance the detection of Iba-1 immunofluorescence in brain tissue damaged by TBI. Iba-1 is a specific marker of microglia in the brain and is known to increase in the area surrounding the injury after TBI (17). However, autofluorescence around the TBI lesion interferes with the imaging effect. Treatment with SBB significantly reduced autofluorescence of damaged brain tissue (by 51.10%), and the specific fluorescence was more prominent compared to untreated tissues (**Figures 5A, B**).

Studies have shown that ethanol can effectively reduce autofluorescence in spinal cord tissue but not in kidney tissue (16, 17). Since the SBB was dissolved in 70% alcohol, we tested whether 70% alcohol itself would reduce background autofluorescence in the TBI model (**Figure 5C**). The background autofluorescence did not decrease significantly after alcohol treatment. Alcohol treatment reduced background fluorescence by 8.18% compared with before treatment (**Figure 5D**). This indicates that SBB treatment was the main reason for effectively shielding autofluorescence.

### Comparison of immunofluorescence of brain tissues damaged by cerebral ischemia before and after SBB treatment

**Figure 6:**
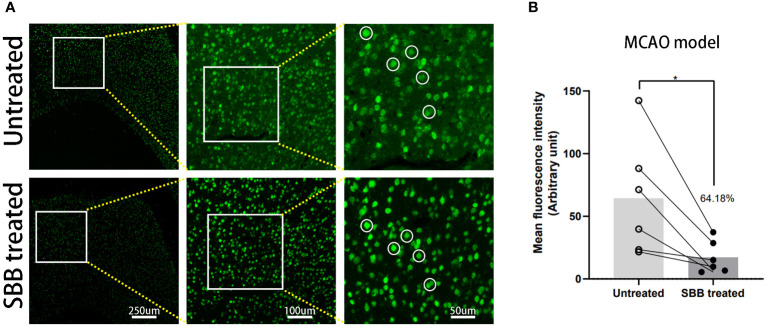
Sudan black B (SBB) blocks autofluorescence of brain tissues damaged by cerebral ischemia in a middle cerebral artery occlusion model. **(A)** Anti-NeuN antibody labeled with Alexa Fluor 488 was used to assess the immunofluorescence of brain tissues damaged by cerebral ischemia in the same brain sections before and after SBB treatment under a fluorescence microscope using the FITC filter. The white circles indicate representative regions of the significant difference in fluorescence signal before and after SBB treatment. **(B)** SBB treatment eliminated tissue autofluorescence by 64.18% compared to before treatment (*p=0.0253; *t*=3.154; paired t-test; n=6 mice/group).

To verify the effect of SBB on paraffin-embedded brain sections, we used brain tissues damaged by cerebral ischemia for immunofluorescence staining. Compared to the untreated control group, SBB treatment significantly reduced the background fluorescence of damaged brain tissues in paraffin-embedded sections and highlighted the specific immunofluorescence signal (**Figure 6A**). Furthermore, quantitative analysis revealed that SBB treatment reduced background fluorescence by 64.18% compared to before treatment (**Figure 6B**).

## Discussion

In this study, we found broad and strong autofluorescence in brain tissues damaged by ICH or TBI with FITC, Tx Red, and DAPI filters. Strong autofluorescence is present immediately around the hemorrhagic or traumatic brain injury site. SBB treatment can effectively reduce background autofluorescence. Furthermore, SBB treatment showed excellent applicability in the fluorescence labeling protocols and immunofluorescence staining with frozen brain sections affected by ICH or TBI. In addition, it offered an efficient blocking effect of autofluorescence of brain tissues damaged by cerebral ischemia in paraffin-embedded brain sections.

The cause of autofluorescence in brain sections is unknown. Unlike other brain disease models, ICH caused by vessel rupture results in massive erythrocyte leakage, releasing fluorescence. In particular, erythrocyte degradation products generate a large amount of protoporphyrin (5), which may be the main reason for autofluorescence immediately around hemorrhagic or traumatic foci. Furthermore, studies have shown that lipofuscin and tissue necrosis produce a large amount of autofluorescence in neural tissues, interfering with fluorescence imaging (25, 26). In a TBI model, damaged brain tissues by external forces cause many necrotic cells in the cerebral cortex, which is suggested to be the leading cause of autofluorescence (27). Therefore, depending on their location and intensity, erythrocytes, necrotic tissue, and lipofuscin are the primary sources of autofluorescence from damaged brain tissues.

Various protocols subtract autofluorescence, including SBB, ammonia-ethanol, sodium borohydride, CuSO_4_, trypan blue, photobleaching, etc. These methods mainly include the following strategies to control autofluorescence: (1) Physical masking of the luminescent group of autofluorescence, (2) dissolving and extracting the substance that can produce autofluorescence, and (3) changing the chemical structure of autofluorescence (27). Ammonia-ethanol can extract and dissolve lipids, eliminating lipid-induced autofluorescence (6). Sodium borohydride reduces autofluorescence by destroying fluorophore molecules through oxidation/reduction reactions (28). Cu^2+^ in CuSO_4_ can remove electrons from autofluorescence compounds and generate a nonfluorescent complex (29). Trypan blue can be uniformly distributed in the cytoplasm and nucleus by permeabilization, and its nonspecific binding fluorescence molecules reduce autofluorescence. The photobleaching method changes the structure of the fluorophore to lose its fluoresce property (3). Trypan blue and CuSO_4_ can reduce autofluorescence, but the appropriate concentration must be determined. Otherwise, the specific fluorescence will be quenched (4, 30). Ultraviolet photobleaching has different effects on the reduction of autofluorescence in various tissues. For example, there was almost no effect on pancreatic tissue but a significant impact on the placenta (3) .

Altering the chemical structure of autofluorescence can reduce autofluorescence. However, it also reduces the intensity of the specific fluorescence and changes the tissue morphology and antigen characteristics. Therefore, it requires carefully considering or exploring the optimal conditions (3, 31).

Earlier studies showed that SBB at a concentration of 0.1% in 70% ethanol treatment was the most effective way to reduce autofluorescence in human brain sections by comparing six autofluorescence removal protocols (32). SBB is a fat-soluble dye that effectively blocks autofluorescence caused by lipofuscin (25), erythrocyte porphyrins (6), and myeloid granules (4). SBB can block the autofluorescence of tissues without interfering with a particular fluorescence at concentrations of 0.1%-0.5% (3, 6, 7, 29). However, higher concentrations of SBB will mask autofluorescence and specific fluorescence signals, making it unable to obtain adequate information, resulting in poor image quality (33). In this study, we demonstrate that 0.15% SBB can effectively reduce autofluorescence while maintaining the specific fluorescence.

To further illustrate the effect of SBB, we analyzed the signal-to-noise ratio of immunofluorescence staining before and after SBB treatment. The signal-to-noise ratio of NeuN staining was effectively increased in the ICH model. SBB treatment provides a reasonable balance between the reduction of total and autofluorescence in tissues and the effective preservation of specific fluorescence signals, resulting in better differentiation of specific and nonspecific fluorescence (33).

The mechanism of action of SBB needs to be clarified. The primary mode of action of the SBB is likely the physical masking of autofluorescence. Studies have shown that SBB has poor solubility in aqueous solution and can cover the hydrophobic region of samples by physical adsorption to give full play to its light absorption capability. The advantage of SBB treatment is that the dye blocks emission peaks of damaged tissue components that generate prominent high autofluorescence (34). Another feature is that the SBB dye can reduce overall autofluorescence so that brain tissue has a homogeneous background and highlights specific fluorescence (4).

Furthermore, unlike other methods, SBB obscures the autofluorescence structure rather than the physical-chemical combination. It can effectively reduce the autofluorescence signal without interfering with specific fluorescence signals (5). However, SBB also has certain limitations. First, although SBB can significantly improve the signal-to-noise ratio of fluorescence imaging, the physical shielding effect of SBB reduces the total fluorescence signal to some extent. Second, SBB treatment adds a treatment step that requires a particular incubation time to reduce tissue autofluorescence.

Another important aspect is the usability of the autofluorescence removal method in different fixation or embedding procedures. For example, ammonia-ethanol can effectively remove the autofluorescence of kidney tissue in frozen sections. However, it does not work in paraffin sections, which is related to the characteristic of ammonia-ethanol in the removal of autofluorescence and the paraffin embedding process (6). Therefore, we use the paraffin embedding method to evaluate the efficacy of SBB in brain tissues damaged by cerebral ischemia. We found that SBB could still remove autofluorescence from paraffin-embedded brain tissues.

Notably, few commercially available quenched autofluorescence-related products, such as True View (Vectorlabs, SP-8500-15), exist. However, TrueView is ineffective against lipofuscin autofluorescence, one of the primary sources of autofluorescence in the brain. As SBB can effectively remove lipofuscin autofluorescence, combining SBB and TrueView may be a promising method for removing additional autofluorescence.

In line with our findings that the application of SBB reduces autofluorescence and improves specific fluorescent signals in brain sections, a recent study uncovered the distribution of angiotensin-converting enzyme 2 in nociceptors in the dorsal root ganglion using SBB to optimize the immunofluorescence protocol (35). Based on our experience and the results of this study, the autofluorescence of multiple wavelengths is generated by the tissues around the lesion after acute brain injury. However, the autofluorescence emission spectra and specific fluorescence signals overlap and interfere with the detection and localization of the specific signals, leading to false-positive results and incorrect interpretations. We showed that SBB could highlight the characteristic fluorescence signal of Cy5.5 by quenching autofluorescence. Therefore, SBB treatment is a highly effective and applicable method for removing autofluorescence, especially in fluorescence imaging experiments of the central nervous system to explore the cellular location of specific proteins.

## Conclusion

In conclusion, we established a feasible and convenient method to optimize the fluorescence imaging protocol using brain sections from mice with acute brain injury. Furthermore, treatment with SBB can effectively quench autofluorescence in brain tissues damaged by acute brain injury. Therefore, it can be used when optimizing the immunofluorescence or fluorescence labeling imaging protocol.

## Data availability statement

The raw data of this article have been deposited in the Mendeley database (DOI: 10.17632/snmyy325k5.1; DOI: 10.17632/3n5bcnwgbs.1), further inquiries can be directed to the corresponding author.

## Ethics statement

The animal study was reviewed and approved by the Animal Ethics Committee of Zhengzhou University.

## Author contributions

SW, XC and JiW conceived the study. SW, XR, JuW, QP, XN, CL, CJ, MZ, ZW, and XF performed the experiments and wrote the manuscript. SW and CS analyzed and reviewed the data, XC and JiW revised and edited the manuscript. All authors contributed to the article and approved the submitted version.
