# Supplementary figures and images for: Blocking autofluorescence in brain tissues affected by ischemic stroke, hemorrhagic stroke, or traumatic brain injury
Source: Front Immunol. 2023 May 29;14:1168292. doi: 10.3389/fimmu.2023.1168292 (PMC10258339; doi:10.3389/fimmu.2023.1168292)

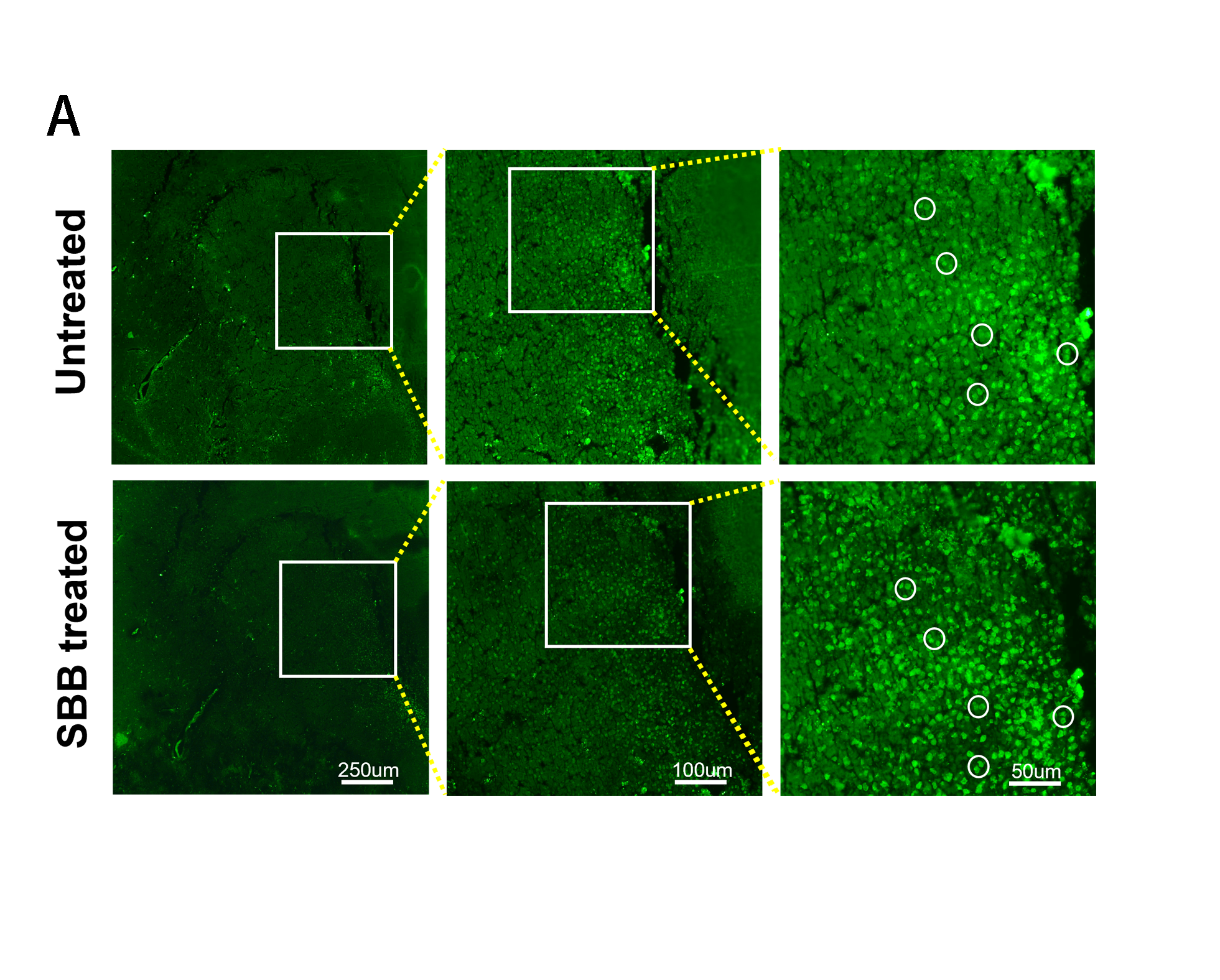

Supplement: Supplementary file 1 [file DataSheet_1.zip › Data Sheet 1/Supplementary Fig.1.tif]

**Normality test**

Figure 3B


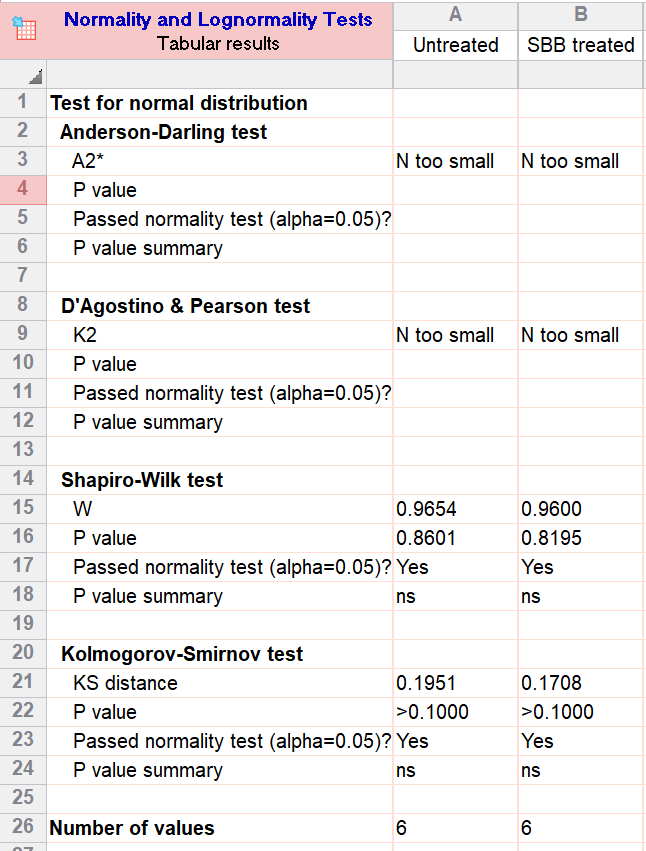


Figure 3D


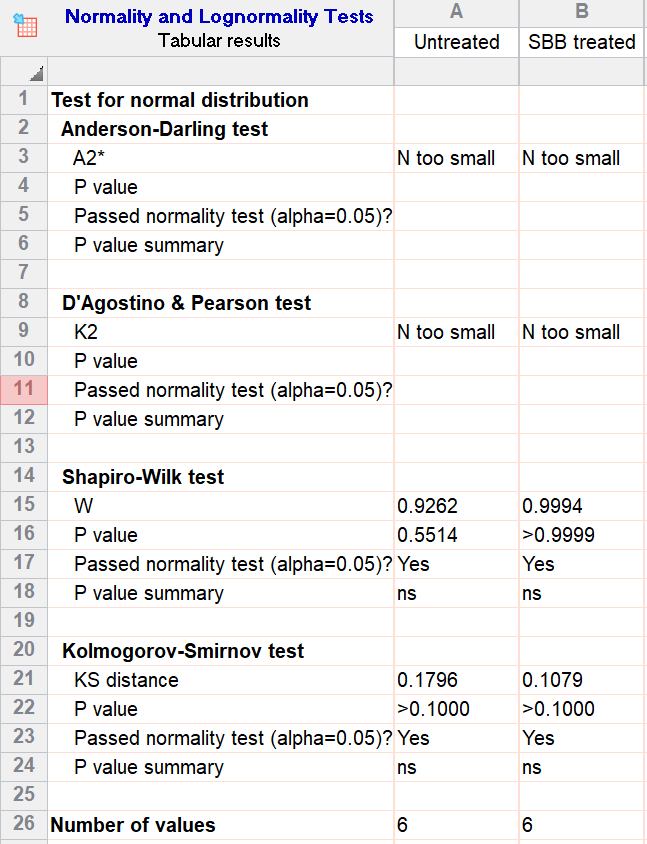


Figure 3E


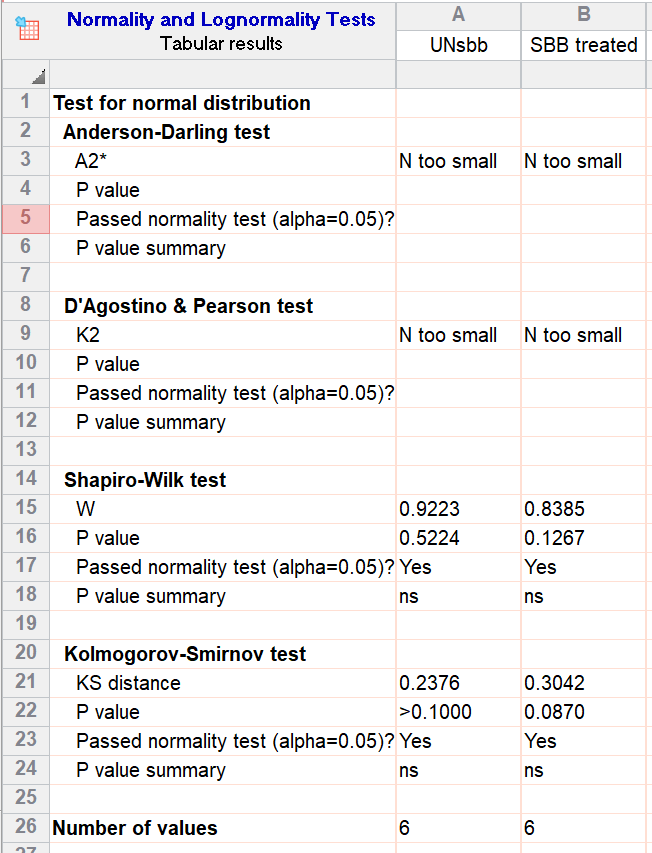


Figure 5B


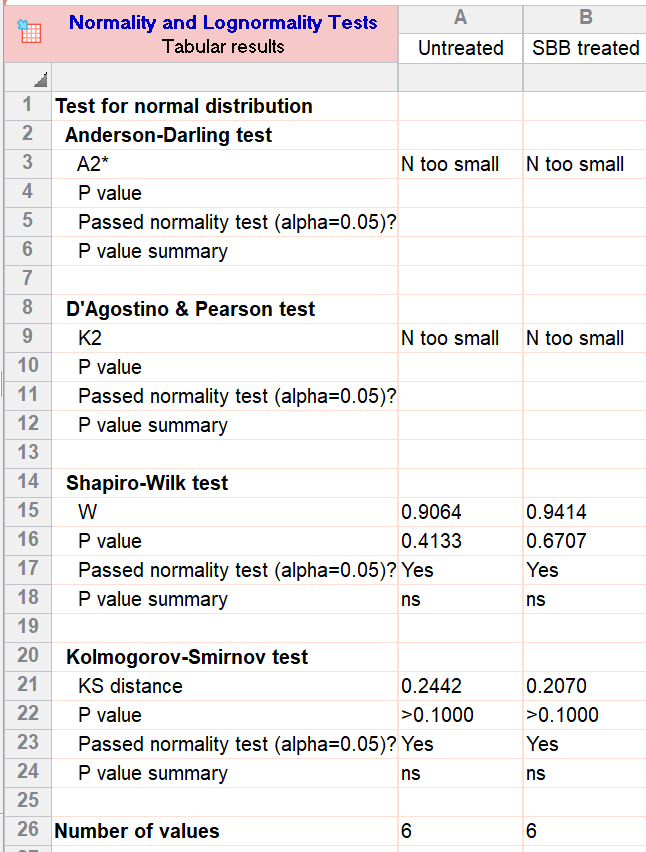


Figure 5D


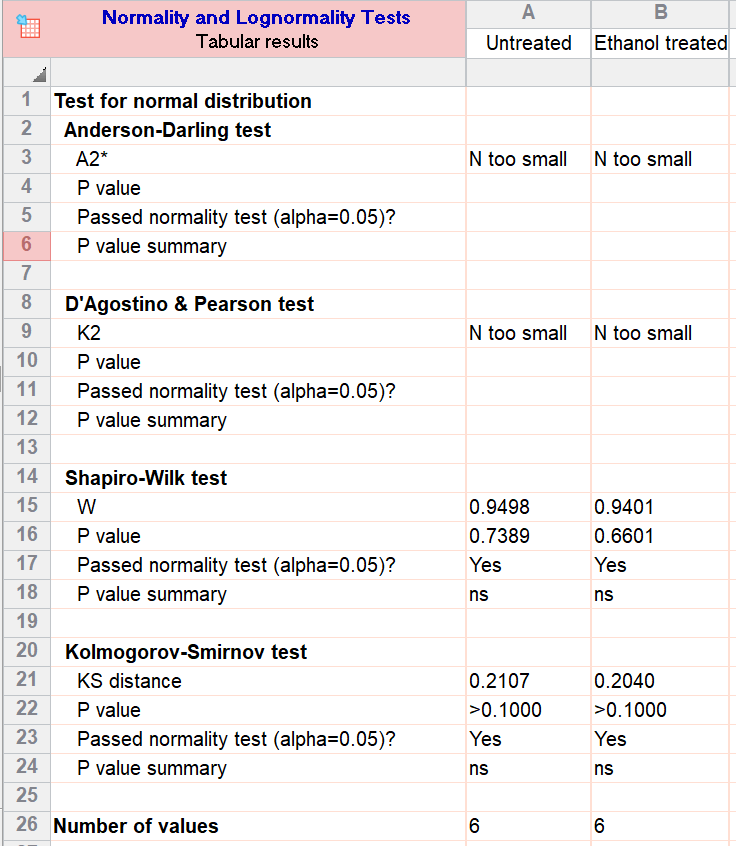


Figure 6B


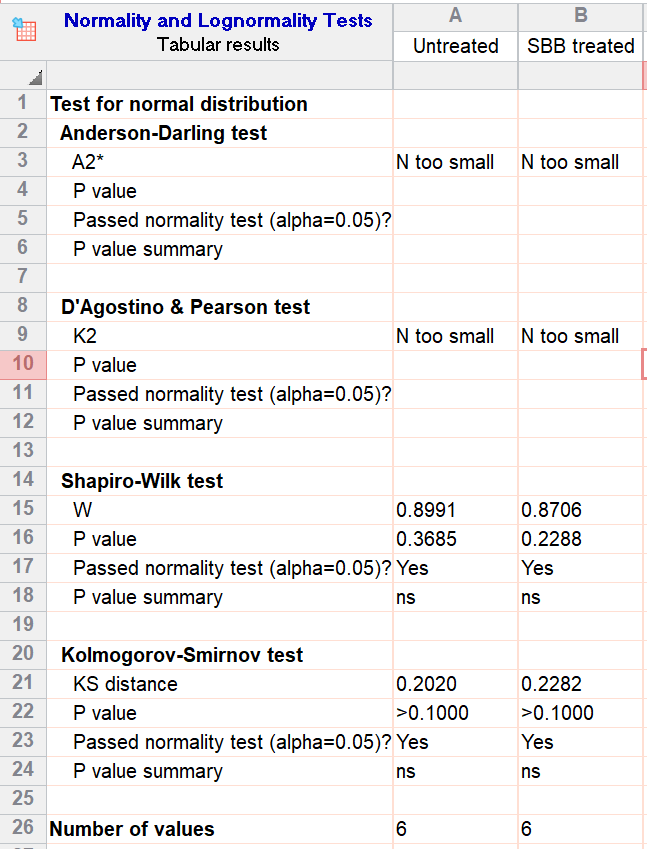

Supplement: Supplementary file 1 [file DataSheet_1.zip › Data Sheet 1/Supplementary materials.docx]
